# MTHFR C677T and postmenopausal breast cancer risk by intakes of one-carbon metabolism nutrients: a nested case-control study

**DOI:** 10.1186/bcr2462

**Published:** 2009-12-23

**Authors:** Sonia S Maruti, Cornelia M Ulrich, Eldon R Jupe, Emily White

**Affiliations:** 1 Public Health Sciences Division, Fred Hutchinson Cancer Research Center, Seattle, WA, 98109-1024 USA; 2 Department of Epidemiology, University of Washington, Seattle, WA, 98195 USA; 3 Biometrics, Hoffmann-La Roche, Inc., 340 Kingsland Street, Nutley, NJ 07110-1199, USA; 4 Research & Development, InterGenetics Incorporated, 655 Research Parkway, Suite 300, Oklahoma City, OK 73104, USA

## Abstract

**Introduction:**

The C677T polymorphism of the methylenetetrahydrofolate reductase (*MTHFR*) gene has been hypothesized to increase breast cancer risk. However, results have been inconsistent, and few studies have reported the association by menopausal status or by intakes of nutrients participating in one-carbon metabolism. Our aims were to investigate whether *MTHFR *C677T was associated with postmenopausal breast cancer risk and whether this relation was modified by intakes of folate, methionine, vitamins B_2_, B_6_, and B_12_, and alcohol.

**Methods:**

We studied 318 incident breast cancer cases and 647 age- and race-matched controls participating in a nested case-control study of postmenopausal women within the VITamins And Lifestyle (VITAL) cohort. Genotyping was conducted for *MTHFR *C677T and dietary and supplemental intakes were ascertained from a validated questionnaire. Adjusted odds ratios (OR) and 95% confidence intervals (CI) were calculated using unconditional logistic regression.

**Results:**

We observed a 62% increased risk of breast cancer among postmenopausal women with the TT genotype (OR = 1.62; 95% CI: 1.05 to 2.48). Women with a higher number of variant T alleles had higher risk of breast cancer (*P *for trend = 0.04). Evidence of effect-modification by intakes of some B vitamins was observed. The most pronounced MTHFR-breast cancer risks were observed among women with the lowest intakes of dietary folate (*P *for interaction = 0.02) and total (diet plus supplemental) vitamin B_6 _(*P *for interaction = 0.01), with no significant increased risks among women with higher intakes.

**Conclusions:**

This study provides support that the *MTHFR *677TT genotype is associated with a moderate increase in risk of postmenopausal breast cancer and that this risk may be attenuated with high intakes of some one-carbon associated nutrients.

## Introduction

Methylenetetrahydrofolate reductase (MTHFR) catalyzes the irreversible reduction of 5,10-methylenetetrahydrofolate to 5-methyltetrahydrofolate, the primary circulating form of folate and methyl donor in DNA methylation. MTHFR is a critical enzyme in one-carbon metabolism, redirecting the pool of folate from DNA synthesis/repair to methylation. It is of interest because aberrations in DNA synthesis, repair, and methylation, have been implicated with cancer risk. The substitution of cytosine (C) with thymine (T) at nucleotide 677 in the *MTHFR *gene is a common polymorphism (C677T) and is correlated with increased thermolability and reduced MTHFR activity [1]. Homozygotes (677TT) have approximately 30% and heterozygotes (677CT) have approximately 65% the activity of homozygous wild-types (677CC), respectively [1,2].

The C677T polymorphism has been studied extensively, yet for breast cancer risk, three recent meta-analyses suggest that the association with the *MTHFR *C677T polymorphism has been largely inconsistent [3-5]. Several reviews suggest that for breast cancer, the relation may vary by menopausal status and also, highlight the importance of examining potential effect of risk modification by nutrients that contribute to or interrupt one-carbon metabolism [3,4]. However, few studies have reported estimates by menopausal status, and most have not examined additional nutrient effect modifiers other than folate or alcohol [3-5]. A further limitation among investigations examining dietary effect modifiers is that most have utilized diet recalled after cancer diagnosis which has the potential for recall bias.

We, therefore, investigated the relationship between the *MTHFR *C677T polymorphism and breast cancer risk among postmenopausal women in a nested case-control study within the VITamins And Lifestyle (VITAL) cohort. We also examined whether this relation was modified by prediagnostic intakes of nutrients involved in one-carbon metabolism (that is, folate, methionine, vitamins B_2_, B_6_, and B_12, _and alcohol). Previously, in the VITAL cohort, we observed a protective association between folate intakes and breast cancer risk [6].

## Materials and methods

### VITAL cohort

The VITAL cohort was principally designed to investigate supplement use and cancer risk. Details have been previously reported [7]. Briefly, men and women were eligible to join the VITAL cohort if they aged 50 and 76 years and living in the western area of Washington State covered by the Surveillance, Epidemiology, and End Results (SEER) cancer registry. A 24-page baseline questionnaire was sent to participants, using names from a commercial mailing list. Data collection occurred from October 2000 to December 2002. Among the 40,339 women who were eligible, 25.6% responded to the baseline questionnaire.

### Selection of cases and controls

Breast cancer cases were identified by linkage to the SEER cancer registry. From baseline to December 30, 2003, 514 breast cancer patients were identified. To form the nested case-control dataset, we excluded women who reported a history of breast cancer (n = 48), had rare breast histologies (that is, sarcoma, phyllodes, or lymphoma, n = 4), did not provide buccal cell samples needed for genotyping (n = 127), or did not complete the breast-cancer risk-factor page of the baseline questionnaire (n = 1), leaving 334 cases. We further excluded 12 women who were not postmenopausal and four women who failed to be genotyped for *MTHFR*. After these criteria, 318 postmenopausal VITAL women who had been diagnosed with incident breast cancer (invasive and *in situ*) remained.

Controls were women who were not diagnosed with any type of cancer since baseline (based on linkage to SEER) and had not reported a history of breast cancer on the baseline questionnaire. There were 36,096 VITAL women without a cancer diagnosis (any type of cancer) since baseline and had never been diagnosed with breast cancer. Of these possible controls, 11,798 were excluded because they did not provide a buccal cell sample, and 185 were excluded because the breast-cancer risk-factor page of the baseline questionnaire was not completed. Two controls for each case were randomly selected from the remaining 24,113 possible (pre- and postmenopausal) controls by frequency matching on age at baseline (in five-year intervals) and race resulting in 668 controls. We also ensured that the follow-up times of the controls (time from baseline to death, a move out of area, or December 31, 2003) were greater than or equal to the follow-up times of the cases (time from baseline to breast cancer diagnosis). Information on deaths and moves out of the area were obtained by linking the VITAL cohort to the Washington State death files and the National Change of Address system. For this current analysis, we excluded 19 controls who were not postmenopausal and two controls whose samples failed to be genotyped for *MTHFR*, leaving 647 postmenopausal controls. This study was approved by the Fred Hutchinson Cancer Research Center Institutional Review Board (IRB), Seattle, Washington. Voluntary return of the questionnaire was considered implied consent.

### Genotyping

DNA was obtained from buccal cells collected from cytobrushes. Women who completed the VITAL baseline questionnaire were mailed a DNA kit containing three sterile cytobrushes, detailed instructions with pictures for use of kit, and a consent form. Genotyping was done by InterGenetics Incorporated (Oklahoma City, OK, USA). Genotyping of *MTHFR *C677T was determined using a microsphere-based, allele-specific primer extension (ASPE) assay followed by analysis on the Luminex 100 flow cytometer (Luminex, Austin, TX, USA) as previously described [8]. DNA was amplified by multiplex PCR using HotStar Taq DNA polymerase (Qiagen Inc., Valencia, CA, USA). To ensure quality control, cases and controls were mixed on genotyping plates, and genotyping was performed with blinding to case-control status. To ensure there was no signal in wells without DNA added, each plate had at least three buffer blanks that were carried through the entire PCR/ASPE/Luminex process. Moreover, at least 5% of the samples were randomly selected and genotyped in duplicate with a concordance rate >99%.

### Effect modifiers and covariates

Information on diet, medical history, reproductive history, lifestyle factors, personal characteristics, and family history of breast cancer was collected at baseline using a 24-page self-administered questionnaire. Women were considered postmenopausal if they had a natural menopause with no periods in the year before baseline, had ever used postmenopausal hormones (PMH), had bilateral oophorectomy, or were 60 years or older at baseline. Because women with a hysterectomy without bilateral oophorectomy cannot report on menopause, they were considered to be postmenopausal if they had ever used hormone therapy or were 55 years or older at baseline.

Intakes of folate, methionine, vitamins B_2_, B_6_, and B_12_, multivitamins and alcohol reported on the baseline questionnaire were examined as potential effect modifiers of the *MTHFR*-breast cancer relationship. Intakes from diet over the past year were determined from a semi-quantitative food frequency questionnaire (FFQ), adapted from the Women's Health Initiative and other studies [9-11]. Participants also reported intakes of multivitamins and individual vitamin supplements, taken singly or as mixtures (for example, *stress*/B-complex, antioxidant mixtures), including questions on years of use in the 10 years before baseline, days per week of use and dose per day. The validity and reliability of supplement reporting in this cohort has been previously examined [12].

We analyzed B vitamins from diet, supplement and diet plus supplement (total) sources. Vitamin B_2 _was not asked as an individual supplement, so only B_2 _as a multivitamin was considered, and therefore supplemental B_2 _was not considered alone. Methionine was determined from diet only. Average daily intake of dietary nutrients was calculated from the FFQ by multiplying the adjusted serving frequency (calculated as frequency times portion size) of each food/beverage item by its nutrient content and summing the nutrient contributions of all foods or beverages. The nutrient database used, the Minnesota Nutrient Data System for Research (University of Minnesota's Nutrition Coordinating Center, Minneapolis, MN, USA) [13], took into account the U.S. mandated folic acid fortification of grain products. Total alcohol intakes were calculated from all reported past-year consumption of red wine, white wine, beer, and liquor/mixed drinks.

Intakes of supplemental folate, other micronutrients, and multivitamins were averaged over 10 years, as previously described [6]. We needed a conversion factor [14] for the calculation of total folate because synthetic folate (folic acid) is more bioavailable than naturally occurring folate (polyglutamates). Thus, total folate, expressed in dietary folate equivalents (DFE), was obtained by first multiplying synthetic folate (supplements and fortified in foods) by a conversion factor of 1.7 and then, adding intakes of natural food folate (μg). To summarize intakes of micronutrients that act as cofactors in the one-carbon pathway, we generated a *one-carbon micronutrient score *by summing the z-scores of total folate, dietary methionine, and total vitamins B_2_, B_6_, B_12 _and then, dividing this score into high (upper median) and low (lower median) for categorical analyses. Women were excluded from the dietary and total (diet plus supplement) nutrient analyses if they did not complete all pages the FFQ or if their reported total energy intake was <600 or >4,000 kcal. Breast cancer risk factors and demographic variables were reported on the baseline questionnaire [6].

### Statistical analyses

We compared baseline characteristics of cases and controls using Wilcoxon signed-rank tests (for continuous variables) or Chi-square tests (for dichotomous variables). Observed genotype frequencies were in Hardy-Weinberg equilibrium (*P *> 0.05). Odds ratios (OR) for breast cancer risk and 95% confidence intervals (CI) were calculated using unconditional logistic regression adjusting for the matching factors of age at baseline (50 to 54 years, 55 to 59, 60 to 64, 65 to 69, 70 to 76) and race (white, other). For analyses of the *MTHFR*-breast cancer association, we assigned the wild-type genotype *MTHFR *677CC as the reference group. Linear trend was calculated by modeling the *MTHFR *genotype (CC, CT, and TT) as one term, ordinally.

Intakes of folate, methionine, vitamins B_2_, B_6_, and B_12_, multivitamins, and alcohol were examined as potential effect modifiers of the *MTHFR*-breast cancer relationship. We dichotomized dietary and total intakes of the B vitamins and methionine by median intakes. Multivitamin intake was divided into some and no intake over the 10 years prior to baseline, and B vitamin consumption from supplements (that is, individual vitamins plus multivitamins) were dichotomized by levels at or above the dose obtained by 10 year daily use of a standard (Centrum^®^) multivitamin versus below that level. Based on previous literature on breast cancer risk [15], and the distribution of alcohol consumption among VITAL women, alcohol was dichotomized into <10 g/d and ≥ 10 g/d intakes (that is, approximately one drink/day). Categorizations of all nutrients were based according to the distribution of intakes among controls. For our examination of effect modification, odds ratios were adjusted for the matching factors of age and race in addition to following standard breast cancer risk factors, as described in the footnote of the last table. To best illustrate the joint effects, we cross-classified women by their *MTHFR *genotype and the dichotomous effect modifier and used a single reference group for the odds ratios. The group hypothesized to have the lowest risk was selected as the reference group. Tests for interaction were performed by entering the product term of the ordinal *MTHFR *variable and dichotomous effect modifier in a multivariable-adjusted model and using Wald statistic to obtain a *P*-value. All statistical analyses were performed using SAS, version 9.1, (SAS Institute Inc., Cary, NC, USA). *P *values < 0.05 were considered statistically significant and all statistical tests were two-sided.

## Results

Participants were on average 64 years of age and mostly Caucasian (Table 1). As expected, cases were significantly more likely than controls to have breast cancer risk factors such as having a prior breast biopsy, having fewer births, using PMH, and drinking more alcohol. Additionally, although not statistically significant, more cases than controls had a first-degree family history, young age at menarche, and were nulliparous. The majority of breast tumors were invasive (n = 253). Among controls, the frequencies of *MTHFR *genotypes were: 677CC (46.5%), 677CT (43.9%), and 677TT (9.6%).

**Table 1:** Characteristics of postmenopausal breast cancer cases and controls, VITAL study *

	**Cases (n = 318)**	**Controls (n = 647)**	***P *value †**
			
Demographics			
Age, years (mean ± SD)	64.4 ± 6.88	64.2 ± 6.86	0.67
White,%	95.0	95.1	0.95
Family history/breast-related procedures			
Mother or sister with breast cancer,%	18.6	15.6	0.25
Mammography in past 2 years,%	93.1	93.7	0.73
Prior breast biopsy,%	28.0	21.8	0.03
Reproductive factors			
Early age at menarche,% <12 years	20.1	18.1	0.44
Nulliparous, %	12.9	9.74	0.14
Age at first birth, years (mean ± SD) ‡	24.1 ± 4.85	23.3 ± 4.39	0.05
Parity, number of births (mean ± SD) ‡	2.60 ± 1.09	2.86 ± 1.21	<0.01
Age at menopause, years (mean ± SD)	47.9 ± 5.37	47.4 ± 5.84	0.37
Ever use of estrogen plus progestin postmenopausal hormones, %	48.1	35.2	<0.01
Lifestyle/anthropomorphic factors			
Height, in (mean ± SD)	64.9 ± 2.65	64.8 ± 2.61	0.36
Baseline BMI, kg/m^2 ^(mean ± SD)	26.9 ± 5.63	27.2 ± 5.61	0.21
Total physical activity, MET-hour/week (mean ± SD)	9.44 ± 11.9	10.1 ± 13.9	0.90
Dietary factors			
Total folate, DFE/day (mean ± SD) §	819 ± 420	856 ± 419	0.15
Dietary methionine, g/day (mean ± SD)	1.43 ± 0.57	1.48 ± 0.61	0.32
Total B_2_, mg/day (mean ± SD) §	2.53 ± 1.21	2.64 ± 1.28	0.16
Total B_6_, mg/day (mean ± SD) §	10.1 ± 30.3	7.97 ± 15.4	0.38
Total B_12_, μg/day (mean ± SD) §	22.5 ± 40.2	18.9 ± 29.3	0.57
Alcohol, g/day (mean ± SD)	5.96 ± 8.91	4.76 ± 9.76	0.03
Multivitamin use, days/week over 10 years (mean ± SD)	3.29 ± 2.90	3.50 ± 2.93	0.25

* Cases and controls were originally 2:1 matched on age and race; the number of cases and controls are not exactly 2:1 because women who were premenopausal or who did not have *MTHFR *genotype data were excluded.

†Wilcoxon rank-sum test for continuous variables and chi-square test for dichotomous variables

‡Among parous women only

§Intake from dietary plus supplemental sources

Postmenopausal women with the *MTHFR *677TT genotype had significantly higher risk of breast cancer (0R = 1.62; 95% CI: 1.05 to 2.48) than 677CC individuals (Table 2). The test for increasing breast cancer risk with increasing number of variant T alleles was significant (*P *for trend = 0.04), although there was no clear excess risk for the heterozygous *MTHFR *677CT genotype (OR = 1.08; 95% CI: 0.81 to 1.43) (Table 2). We observed an increased breast cancer risk with *MTHFR *677TT even after restricting to invasive breast cancer cases (RR = 1.65; 95% CI: 1.03 to 2.63) and when restricting to Caucasians (RR = 1.59; 95% CI: 1.03 to 2.46 for 302 cases) (data not shown).

**Table 2:** Odds ratios (OR) for postmenopausal breast cancer by *MTHFR *C677T genotype, VITAL study

	Cases, n	Controls, n	OR (95% CI)
CC	133	301	1.00
CT	139	284	1.08 (0.81 to 1.43)
TT	46	62	1.62 (1.05 to 2.48)
P for trend			0.04

* Adjusted for age in five year-age categories and race (white, other).

† *P *for trend calculated by modelling the *MTHFR*. genotypes CC, CT, and TT as one term, ordinally.

We next examined whether the *MTHFR*-breast cancer association was modified by intakes of nutrients involved in one-carbon metabolism (Table 3). In general, elevated risks of breast cancer were observed among individuals with the 677TT genotype in the lower nutrient intake groups, and statistically significant trends with genotype were seen in these groups. Furthermore, odds ratios appeared stronger among groups defined by low intake of nutrients from total sources than from diet or supplements alone. The similarity of these results across nutrients is due, in part, to the high correlation between intakes of total folate and vitamins B_2_, B_6_, and B_12 _(range of correlations between nutrients (r): 0.60 to 0.79). However, only two of these results showed a statistically significant interaction: the risk of breast cancer among women with the *MTHFR *677TT genotype was significantly higher among women with low intakes of dietary folate (*P *for interaction = 0.02) and total B_6 _(*P *for interaction = 0.01) than those with higher intakes. Results for total vitamin B_6 _were particularly striking. Among women with high B_6 _intake, there was no MTHFR-breast cancer association. However, among women with low B_6 _intake, the TT genotype was associated with an approximate four-fold risk (OR = 4.03 = 4.47/1.11). Alcohol intake did not appear to modify the exposure-disease association (*P *for interaction = 0.22), but few VITAL women drank ≥ 10 g/d of alcohol.

**Table 3:** Joint association of *MTHFR *C677T genotype and nutrient intakes on postmenopausal breast cancer risk, VITAL study *,†

	*MTHFR *C677T genotype							
	CC		CT		TT			
					
	Cases/controls	Multivariate OR (95% CI)	Cases/controls	Multivariate OR (95% CI)	Cases/controls	Multivariate OR (95% CI)	P for trend ‡	P for interaction §
**Multivitamin use**								
Some	92/218	1.00	108/213	1.20 (0.82-1.75)	29/51	1.46 (0.83-2.56)	0.24	
None	41/83	1.24 (0.75-2.03)	31/71	0.90 (0.52-1.59)	17/11	6.24 (2.37-16.4)	0.02	0.27
**Folate**								
Dietary folate (μg/day)								
≥ 224 (median, high)	79/128	1.00	68/135	0.85 (0.55-1.30)	20/27	1.29 (0.66-2.52)	0.86	
<224	43/139	0.41 (0.25-0.67)	55/126	0.60 (0.37-0.98)	26/27	1.31 (0.66-2.63)	<0.01	0.02
Supplemental folate (μg/day) #								
≥ 400	48/129	1.00	58/119	1.28 (0.80-2.06)	13/26	1.17 (0.54-2.54)	0.53	
<400	85/172	1.34(0.87-2.07)	81/165	1.39 (0.89-2.15)	33/36	2.69 (1.48-4.88)	0.05	0.83
Total folate (DFE/day)								
≥ 850 (median, high)	54/132	1.00	65/127	1.26 (0.79-2.01)	13/29	1.03 (0.48-2.22)	0.74	
<850	68/134	1.32 (0.83-2.09)	57/130	1.19 (0.74-1.91)	32/23	4.09 (2.08-8.04)	0.01	0.19
**Methionine**								
Dietary methionine (g/day)								
≥ 1.38 (median, high)	60/133	1.00	56/135	0.91 (0.58-1.44)	19/23	1.89 (0.91-3.89)	0.29	
<1.38	62/134	1.07 (0.65-1.77)	67/126	1.29 (0.78-2.13)	27/31	2.10 (1.07-4.14)	0.04	0.55
**Vitamin B_2_**								
Dietary vitamin B_2 _(mg/day)								
≥ 1.81 (median, high)	63/127	1.00	59/133	0.92 (0.58-1.44)	18/29	1.23 (0.62-2.45)	0.72	
<1.81	59/140	0.85 (0.52-1.37)	64/128	1.03 (0.63-1.68)	28/25	2.53 (1.25-5.12)	<0.01	0.06
Total vitamin B_2 _(mg/day)								
≥ 2.70 (median, high)	59/125	1.00	60/139	0.94 (0.59-1.48)	16/26	1.27 (0.61-2.64)	0.96	
<2.70	63/142	1.02(0.64-1.63)	63/122	1.23 (0.76-1.98)	39/28	2.73 (1.40-5.33)	0.02	0.10
**Vitamin B_6_**								
Dietary vitamin B_6 _(mg/day)								
≥ 1.61 (median, high)	59/129	1.00	54/132	0.93 (0.58-1.48)	16/31	1.20 (0.59-2.45)	0.97	
<1.61	63/138	1.08 (0.66-1.77)	69/129	1.26 (0.77-2.07)	30/23	3.23 (1.58-6.61)	<0.01	0.07
Supplemental vitamin B_6 _(mg/day) #								
≥ 2.00	49/108	1.00	55/103	1.17 (0.72-1.90)	10/25	0.69 (0.30-1.60)	0.93	
<2.00	84/192	0.87 (0.57-1.34)	83/177	0.97 (0.62-1.50)	36/35	2.31 (1.28-4.15)	<0.01	0.11
Total vitamin B_6 _(mg/day)								
≥ 3.32 (median, high)	57/132	1.00	62/130	1.11 (0.70-1.76)	12/32	0.72 (0.33-1.56)	0.83	
<3.32	65/135	1.11 (0.70-1.75)	61/131	1.11 (0.70-1.76)	34/22	4.47 (2.28-8.75)	<0.01	0.01
**Vitamin B_12_**								
Dietary vitamin B_12 _(μg/day)								
≥ 5.30 (median, high)	52/131	1.00	57/133	1.13 (0.70-1.80)	19/26	2.01 (0.99-4.11)	0.15	
<5.30	70/136	1.38 (0.85-2.22)	66/128	1.38 (0.85-2.26)	27/28	2.58 (1.31-5.08)	0.10	0.85
Supplemental vitamin B_12 _(μg/day) #								
≥ 6.00	56/120	1.00	69/109	1.30 (0.83-2.05)	14/25	1.06 (0.50-2.25)	0.44	
<6.00	76/180	0.81 (0.53-1.24)	70/171	0.83 (0.54-1.29)	29/34	1.72 (0.94-3.15)	0.04	0.65
Total vitamin B_12 _(μg/day)								
≥ 11.4 (median, high)	60/130	1.00	69/131	1.17 (0.75-1.83)	17/33	1.15 (0.57-2.33)	0.48	
<11.4	62/137	0.98 (0.62-1.53)	54/129	0.92 (0.58-1.47)	29/21	3.06 (1.55-6.04)	<0.01	0.17
Micronutrient score**								
High (median)	57/129	1.00	62/134	1.09 (0.69-1.73)	13/27	1.05 (0.48-2.28)	0.96	
Low	65/138	1.12 (0.70-1.80)	61/127	1.15 (0.71-1.87)	33/27	3.20 (1.66-6.19)	<0.01	0.10
**Total alcohol (g/d)**								
<10.0 (low)	100/254	1.00	105/229	1.15 (0.81-1.65)	38/53	2.12 (1.26-3.56)	0.01	
≥ 10.0	32/42	1.80 (1.02-3.15)	30/48	1.34 (0.76-2.38)	8/8	2.32 (0.79-6.85)	0.84	0.22

* All analyses adjusted for the following:age (50-54 years, 55-59, 60-64, 65-69, 70-76), race (white, other), family history of breast cancer (no, 1 affected mother or sister, ≥ 2 affected mother or sister(s)), mammography within two years preceding baseline(no, yes), history of breast biopsy (no, yes), age at menarche (≤ 11 years,12,13, ≥ 14), age at first birth (nulliparous, ≤ 19 years, 20-24, 25-34, ≥ 35), age at menopause (≤ 44 years,45-49, ≥ 50), years of combined estrogen and progestin postmenopausal hormones (PMH, never or <1 years,1-4,5-9, ≥ 10), height (<62 in, 62-<65, 65-<68, ≥ 68), body mass index (BMI) (<25 kg/m^2^,25-<30, ≥ 30), total physical activity (none, 1-3 tertiles of MET-hours/week), total energy intake (kcal/day), and for non-alcohol exposures, past-year alcohol intake (<1.5 g/day, 1.5-4.9, 5.0-9.9, ≥ 10). Energy was added to the models for all dietary and total nutrient exposures.

† Case/control numbers do not add up to total due to missing data on nutrient intakes

‡ P for trend testing trend for *MTHFR *genotypes by strata of nutrient/alcohol intakes and calculated by modelling the *MTHFR *genotypes (CC, CT, and TT) as one term, ordinally

§P for interaction testing whether the association between *MTHFR *and breast cancer varies by nutrient/alcohol intake

Median intakes, based on distribution of nutrient intake from controls

# Intakes of all supplements over 10 years; cutpoints represent amount in a 10 year daily use of standard multivitamin or greater

** Micronutrient score computed by summing the z-scores of folate, methionine, and vitamins B_2_, B_6_, B_12 _and then, dividing this sum into high (upper median) and low (lower median) groups.

## Discussion

In this moderate-sized, nested case-control study, we observed a 62% increase risk of breast cancer among postmenopausal women with the TT genotype. The most pronounced risks were observed among individuals with the TT genotype and lowest intakes of folate and vitamin B_6_.

Results from 26 case-control studies [4,16-40] investigating *MTHFR *C677T and breast cancer risk have been inconsistent. Our results are consistent with two [18,19] of nine studies [17-20,22,26,27,29,31] reporting separate estimates for postmenopausal women. Ericson et al observed a significant 34% increase in breast cancer risk among postmenopausal women in Sweden with CT and TT genotypes compared to wild-type in a nested case-control study of the Malmo Diet and Cancer cohort [19]. Suzuki reported a significant 83% increased breast cancer risk among postmenopausal Japanese women with the TT genotype compared to wild-type [18]. Among 10 studies reporting estimates for premenopausal women [16-19,22,26,27,29-31], three have reported significant positive associations, ranging from a 64% to a 2.8-fold increased risk among CT and/or TT individuals [16,17,30]. Two studies [23,32] observed statistically significant increased risks among pre- and postmenopausal women combined. Other investigations have not reported significant associations. Differences in results may be due to variation between populations with regards to prevalence of polymorphisms in genes related to one-carbon metabolism, intakes of nutrients, and/or risk factors for breast cancer. Several studies reporting no association had <150 breast cancer cases [4,24,25,33,34,36,37,39], and thus, may have been too small to detect an association. Our study tended to have a larger population of postmenopausal breast cancer patients, and thus, may have had more power to detect an effect.

Eight studies, to-date, have examined interactions between *MTHFR *C677T and nutrients, including alcohol [17-23,25]. Among these, only a case-control study of Brazilian women (458 age-matched pairs) observed statistically significant gene-diet interactions [22]. However, the folate results were opposite than expected; a significantly reduced risk of breast cancer was observed among TT and CT individuals with the lowest intakes of dietary folate. Major limitations were that diet was collected after breast cancer diagnosis and recall of diet was poor. While the tests of interaction were not significant, three studies observed increased MTHFR-breast cancer risks with low folate intakes which are in line with our results [18,21,23].

Our results are biologically plausible. The TT genotype is associated with approximately 65% less activity than wild-type [1,2]. Reduced MTHFR activity among 677TT individuals may increase cancer risk by leading to lowered availability of 5-methyltetrahydrofolate and subsequently, impaired DNA methylation. DNA methylation plays a critical role in gene expression and the maintenance of genomic stability [41,42], and dysregulation of methylation patterns have been implicated with carcinogenesis [43,44]. Furthermore, our results suggest a more pronounced risk of breast cancer among 677TT individuals when intakes of nutrients associated with one-carbon metabolism are comparatively low. This finding is consistent with multiple studies showing increases in risk of colorectal adenomas or cancer under a low one-carbon status [45,46]. Furthermore, our results are supported by other reports suggesting that when folate levels are low, the 677TT genotype is associated with higher levels of homocysteine, lower levels of methylated folate, and reductions in genomic DNA methylation [47,48]. Our previous study in VITAL and other reports suggest that folate intake lowers breast cancer risk [6]. Reasons for not being able to replicate the earlier VITAL cohort findings may be that this nested case-control study had approximately three-years shorter follow-up and fewer cases (334 versus 743 cases in the cohort study publication). Vitamin B_6 _has an important role in one-carbon metabolism in that it acts as a cofactor for methionine synthesis and is a coenzyme of serine hydroxymethyltransferase, which is involved in nucleotide synthesis. We did not observe an overall statistically significant association between B_6 _intake and breast cancer risk, previously [6]. However, it is possible that intakes below a certain threshold may make 677TT individuals more susceptible.

Our study has some limitations. First, we did not have data on other *MTHFR *polymorphisms, such as A1298C and G1793A. However, data suggest that C677T is the major genetic determinant of MTHFR activity [49]. Second, the high correlation between nutrient intakes (r ≥ 0.60) made it difficult to separately examine effect modification of individual nutrients. Lastly, nutrient and supplement intakes were based on self-report; however, because this information was collected prior to diagnosis, any misclassification would have been non-differential and would most likely have attenuated the associations. Another source of measurement error, apart from inaccuracies in self-report, is that the folate content of food changed over time. Food manufacturers began fortifying grain products starting in 1996 to 1998 in response to U.S. governmental regulations, a few years before the VITAL baseline questionnaire (2000 to 2002). Thus, the high levels of intake in this population represent the post-fortification period, while intake earlier during the pre-fortification period may be more predictive of breast cancer risk. Since low folate intake is of most interest in MTHR effect modification, use of post-fortification folate values may have weakened our ability to detect effect modification by low folate intakes.

Our study adds to the literature by being the first study, to our knowledge, examining effect modification of *MTHFR *by multiple nutrients involved in the one-carbon metabolism pathway; data on supplement use also allowed for analysis by nutrient type (diet, supplement, and total). This study also adds to current knowledge regarding the association of *MTHFR *among postmenopausal women. An advantage of this study's prospective design is that it avoids recall bias for the recall of nutrients. An additional strength is that we had a broad range of information on possible confounders for adjustment. Also, selection bias is highly unlikely, because this was a prospective study and participants would not have known their future breast cancer status when deciding to give buccal cells or complete the breast cancer risk questions.

## Conclusions

In summary, this study provides support that the *MTHFR *677TT genotype is associated with a moderately increased risk of postmenopausal breast cancer and with a substantial increase among women with low intakes of folate and vitamin B_6_. From a public health standpoint, these results are of interest in that they suggest that the increased risk associated with the TT genotype may be attenuated with intakes of some one-carbon associated nutrients.

## Abbreviations

ASPE: allele-specific primer extension; BMI: body mass index; CI: confidence interval; DNA: deoxyribonucleic acid; FFQ: food frequency questionnaire; IRB: Institutional Review Board; kcal: kilocalorie; MET: metabolic equivalent; MTHFR: methylenetetrahydrofolate reductase; PMH: postmenopausal hormones; OR: odds ratio; PCR: polymerase chain reaction; SD: standard deviation; SEER: Surveillance, Epidemiology, and End Results; VITAL: VITamins And Lifestyle.

## Competing interests

ERJ is a salaried employee of and holds stock options in InterGenetics Incorporated, but is not a major shareholder. A grant from InterGenetics Incorporated provided partial support for genotyping analyses. The other authors declare that they have no competing interests.

## Authors' contributions

SSM planned the data analysis, carried out all statistical analyses, interpreted the results, and drafted the manuscript. CMU contributed to the interpretation of the data and revised the manuscript for important intellectual content. ERJ directed the DNA isolation and the acquisition and finalization of the genotyping studies and revised the manuscript. EW conceived of the VITAL study, obtained funding, participated in the study's design and coordination, contributed to the interpretation of results, and revised the manuscript for important intellectual content. All authors read and approved the final manuscript.

## References

[B1] Frosst P Blom HJ Milos R Goyette P Sheppard CA Matthews RG Boers GJ den Heijer M Kluijtmans LA Heuvel LP van den Rozen R A candidate genetic risk factor for vascular disease: a common mutation in methylenetetrahydrofolate reductase Nature Genetics 1995 10 111 113 10.1038/ng0595-111 7647779

[B2] Weisberg IS Jacques PF Selhub J Bostom AG Chen Z Curtis Ellison R Eckfeldt JH Rozen R The 1298A-->C polymorphism in methylenetetrahydrofolate reductase (MTHFR): in vitro expression and association with homocysteine Atherosclerosis 2001 156 409 415 10.1016/S0021-9150(00)00671-7 11395038

[B3] Zintzaras E Methylenetetrahydrofolate reductase gene and susceptibility to breast cancer: a meta-analysis Clinical Genetics 2006 69 327 336 10.1111/j.1399-0004.2006.00605.x 16630166

[B4] Macis D Maisonneuve P Johansson H Bonanni B Botteri E Iodice S Santillo B Penco S Gucciardo G D'Aiuto G Rosselli Del Turco M Amadori M Costa A Decensi A Methylenetetrahydrofolate reductase (MTHFR) and breast cancer risk: a nested-case-control study and a pooled meta-analysis Breast Cancer Res Treat 2007 106 263 271 10.1007/s10549-006-9491-6 17260091

[B5] Lewis SJ Harbord RM Harris R Smith GD Meta-analyses of observational and genetic association studies of folate intakes or levels and breast cancer risk Journal of the National Cancer Institute 2006 98 1607 1622 17105984 10.1093/jnci/djj440

[B6] Maruti SS Ulrich CM White E Folate and one-carbon metabolism nutrients from supplements and diet in relation to breast cancer risk The American Journal of Clinical Nutrition 2009 89 624 633 10.3945/ajcn.2008.26568 19116331 PMC2647765

[B7] White E Patterson RE Kristal AR Thornquist M King I Shattuck AL Evans I Satia-Abouta J Littman AJ Potter JD VITamins And Lifestyle cohort study: study design and characteristics of supplement users American Journal of Epidemiology 2004 159 83 93 10.1093/aje/kwh010 14693663

[B8] Diergaarde B Potter JD Jupe ER Manjeshwar S Shimasaki CD Pugh TW Defreese DC Gramling BA Evans I White E Polymorphisms in genes involved in sex hormone metabolism, estrogen plus progestin hormone therapy use, and risk of postmenopausal breast cancer Cancer Epidemiol Biomarkers Prev 2008 17 1751 1759 18628428 10.1158/1055-9965.EPI-08-0168 PMC2732341

[B9] Patterson RE Neuhouser ML White E Kristal AR Potter JD Measurement error from assessing use of vitamin supplements at one point in time Epidemiology (Cambridge, Mass) 1998 9 567 569 9730039

[B10] Kristal AR Feng Z Coates RJ Oberman A George V Associations of race/ethnicity, education, and dietary intervention with the validity and reliability of a food frequency questionnaire: the Women's Health Trial Feasibility Study in Minority Populations American Journal of Epidemiology 1997 146 856 869 9384206 10.1093/oxfordjournals.aje.a009203

[B11] Patterson RE Kristal AR Tinker LF Carter RA Bolton MP Agurs-Collins T Measurement characteristics of the Women's Health Initiative food frequency questionnaire Annals of Epidemiology 1999 9 178 187 10.1016/S1047-2797(98)00055-6 10192650

[B12] Satia-Abouta J Patterson RE King IB Stratton KL Shattuck AL Kristal AR Potter JD Thornquist MD White E Reliability and validity of self-report of vitamin and mineral supplement use in the vitamins and lifestyle study American Journal of Epidemiology 2003 157 944 954 10.1093/aje/kwg039 12746248

[B13] Schakel SF Buzzard IM Gebhardt SE Procedures for estimating nutrient values for food composition databases J Food Composition Anal 1997 10 102 114 10.1006/jfca.1997.0527

[B14] Dietary reference Intakes for thiamin, riboflavin, niacin, vitamin B6, folate, vitamin B12, pantothenic acid, biotin, and choline 2000 Washington DC: Institute of Medicine, National Academy of Sciences 210 23193625

[B15] Smith-Warner SA Spiegelman D Yaun SS Brandt PA van den Folsom AR Goldbohm RA Graham S Holmberg L Howe GR Marshall JR Miller AB Potter JD Speizer FE Willett WC Wolk A Hunter DJ Alcohol and breast cancer in women: a pooled analysis of cohort studies JAMA 1998 279 535 540 10.1001/jama.279.7.535 9480365

[B16] Campbell IG Baxter SW Eccles DM Choong DY Methylenetetrahydrofolate reductase polymorphism and susceptibility to breast cancer Breast Cancer Res 2002 4 R14 12473175 10.1186/bcr457 PMC137931

[B17] Semenza JC Delfino RJ Ziogas A Anton-Culver H Breast cancer risk and methylenetetrahydrofolate reductase polymorphism Breast Cancer Res Treat 2003 77 217 223 10.1023/A:1021843019755 12602921

[B18] Suzuki T Matsuo K Hirose K Hiraki A Kawase T Watanabe M Yamashita T Iwata H Tajima K One-carbon metabolism-related gene polymorphisms and risk of breast cancer Carcinogenesis 2008 29 356 362 10.1093/carcin/bgm295 18174236

[B19] Ericson U Sonestedt E Ivarsson MI Gullberg B Carlson J Olsson H Wirfalt E Folate intake, methylenetetrahydrofolate reductase polymorphisms, and breast cancer risk in women from the Malmo Diet and Cancer cohort Cancer Epidemiol Biomarkers Prev 2009 18 1101 1110 10.1158/1055-9965.EPI-08-0401 19336565

[B20] Stevens VL McCullough ML Pavluck AL Talbot JT Feigelson HS Thun MJ Calle EE Association of polymorphisms in one-carbon metabolism genes and postmenopausal breast cancer incidence Cancer Epidemiol Biomarkers Prev 2007 16 1140 1147 10.1158/1055-9965.EPI-06-1037 17548676

[B21] Shrubsole MJ Gao YT Cai Q Shu XO Dai Q Hebert JR Jin F Zheng W MTHFR polymorphisms, dietary folate intake, and breast cancer risk: results from the Shanghai Breast Cancer Study Cancer Epidemiol Biomarkers Prev 2004 13 190 196 10.1158/1055-9965.EPI-03-0273 14973091

[B22] Ma E Iwasaki M Junko I Hamada GS Nishimoto IN Carvalho SM Motola J Jr Laginha FM Tsugane S Dietary intake of folate, vitamin B6, and vitamin B12, genetic polymorphism of related enzymes, and risk of breast cancer: a case-control study in Brazilian women BMC Cancer 2009 9 122 19389261 10.1186/1471-2407-9-122 PMC2684745

[B23] Chen J Gammon MD Chan W Palomeque C Wetmur JG Kabat GC Teitelbaum SL Britton JA Terry MB Neugut AI Santella RM One-carbon metabolism, MTHFR polymorphisms, and risk of breast cancer Cancer Research 2005 65 1606 1614 10.1158/0008-5472.CAN-04-2630 15735051

[B24] Beilby J Ingram D Hahnel R Rossi E Reduced breast cancer risk with increasing serum folate in a case-control study of the C677T genotype of the methylenetetrahydrofolate reductase gene Eur J Cancer 2004 40 1250 1254 10.1016/j.ejca.2004.01.026 15110890

[B25] Sharp L Little J Schofield AC Pavlidou E Cotton SC Miedzybrodzka Z Baird JO Haites NE Heys SD Grubb DA Folate and breast cancer: the role of polymorphisms in methylenetetrahydrofolate reductase (MTHFR) Cancer Letters 2002 181 65 71 10.1016/S0304-3835(02)00030-7 12430180

[B26] Kotsopoulos J Zhang WW Zhang S McCready D Trudeau M Zhang P Sun P Narod SA Polymorphisms in folate metabolizing enzymes and transport proteins and the risk of breast cancer Breast Cancer Res Treat 2008 112 585 593 10.1007/s10549-008-9895-6 18204969

[B27] Le Marchand L Haiman CA Wilkens LR Kolonel LN Henderson BE MTHFR polymorphisms, diet, HRT, and breast cancer risk: the multiethnic cohort study Cancer Epidemiol Biomarkers Prev 2004 13 2071 2077 15598763

[B28] Langsenlehner U Krippl P Renner W Yazdani-Biuki B Wolf G Wascher TC Paulweber B Weitzer W Samonigg H The common 677C>T gene polymorphism of methylenetetrahydrofolate reductase gene is not associated with breast cancer risk Breast Cancer Res Treat 2003 81 169 172 10.1023/A:1025752420309 14572159

[B29] Justenhoven C Hamann U Pierl CB Rabstein S Pesch B Harth V Baisch C Vollmert C Illig T Bruning T Ko Y Brauch H One-carbon metabolism and breast cancer risk: no association of MTHFR, MTR, and TYMS polymorphisms in the GENICA study from Germany Cancer Epidemiol Biomarkers Prev 2005 14 3015 3018 10.1158/1055-9965.EPI-05-0592 16365030

[B30] Ergul E Sazci A Utkan Z Canturk NZ Polymorphisms in the MTHFR gene are associated with breast cancer Tumour Biol 2003 24 286 290 10.1159/000076460 15004488

[B31] Lee SA Kang D Nishio H Lee MJ Kim DH Han W Yoo KY Ahn SH Choe KJ Hirvonen A Noh DY Methylenetetrahydrofolate reductase polymorphism, diet, and breast cancer in Korean women Experimental & Molecular Medicine 2004 36 116 121 15150439 10.1038/emm.2004.17

[B32] Jakubowska A Gronwald J Menkiszak J Gorski B Huzarski T Byrski T Edler L Lubinski J Scott RJ Hamann U Methylenetetrahydrofolate reductase polymorphisms modify BRCA1-associated breast and ovarian cancer risks Breast Cancer Res Treat 2007 104 299 308 10.1007/s10549-006-9417-3 17063264

[B33] Hekim N Ergen A Yaylim I Yilmaz H Zeybek U Ozturk O Isbir T No association between methylenetetrahydrofolate reductase C677T polymorphism and breast cancer Cell Biochem and Funct 2007 25 115 117 10.1002/cbf.1274 16134079

[B34] Kalemi TG Lambropoulos AF Gueorguiev M Chrisafi S Papazisis KT Kotsis A The association of p53 mutations and p53 codon 72, Her 2 codon 655 and MTHFR C677T polymorphisms with breast cancer in Northern Greece Cancer Letters 2005 222 57 65 10.1016/j.canlet.2004.11.025 15837541

[B35] Chou YC Wu MH Yu JC Lee MS Yang T Shih HL Wu TY Sun CA Genetic polymorphisms of the methylenetetrahydrofolate reductase gene, plasma folate levels and breast cancer susceptibility: a case-control study in Taiwan Carcinogenesis 2006 27 2295 2300 10.1093/carcin/bgl108 16777985

[B36] Langsenlehner T Renner W Yazdani-Biuki B Langsenlehner U Methylenetetrahydrofolate reductase (MTHFR) and breast cancer risk: a nested-case-control study and a pooled meta-analysis Breast Cancer Res Treat 2007 107 459 460 10.1007/s10549-007-9564-1 17453338

[B37] Reljic A Simundic AM Topic E Nikolac N Justinic D Stefanovic M The methylenetetrahydrofolate reductase (MTHFR) C677T polymorphism and cancer risk: the Croatian case-control study Clinical Biochemistry 2007 40 981 985 10.1016/j.clinbiochem.2007.05.005 17573062

[B38] Inoue M Robien K Wang R Berg DJ Van Den Koh WP Yu MC Green tea intake, MTHFR/TYMS genotype and breast cancer risk: the Singapore Chinese Health Study Carcinogenesis 2008 29 1967 1972 18669903 10.1093/carcin/bgn177 PMC2574755

[B39] Yu CP Wu MH Chou YC Yang T You SL Chen CJ Sun CA Breast cancer risk associated with multigenotypic polymorphisms in folate-metabolizing genes: a nested case-control study in Taiwan Anticancer Research 2007 27 1727 1732 17595805

[B40] Cheng CW Yu JC Huang CS Shieh JC Fu YP Wang HW Wu PE Shen CY Polymorphism of cytosolic serine hydroxymethyltransferase, estrogen and breast cancer risk among Chinese women in Taiwan Breast Cancer Res Treat 2008 111 145 155 10.1007/s10549-007-9754-x 17896178

[B41] Kundu TK Rao MR CpG islands in chromatin organization and gene expression Journal of Biochemistry 1999 125 217 222 9990116 10.1093/oxfordjournals.jbchem.a022276

[B42] Lengauer C Kinzler KW Vogelstein B DNA methylation and genetic instability in colorectal cancer cells Proceedings of the National Academy of Sciences of the United States of America 1997 94 2545 2550 9122232 10.1073/pnas.94.6.2545 PMC20125

[B43] Jones PA Baylin SB The fundamental role of epigenetic events in cancer Nature Reviews 2002 3 415 428 12042769 10.1038/nrg816

[B44] Bariol C Suter C Cheong K Ku SL Meagher A Hawkins N Ward R The relationship between hypomethylation and CpG island methylation in colorectal neoplasia The American Journal of Pathology 2003 162 1361 1371 12651628 10.1016/S0002-9440(10)63932-6 PMC1851239

[B45] Ulrich CM Nutrigenetics in cancer research--folate metabolism and colorectal cancer The Journal of Nutrition 2005 135 2698 2702 16251633 10.1093/jn/135.11.2698

[B46] Ulrich CM Kampman E Bigler J Schwartz SM Chen C Bostick R Fosdick L Beresford SA Yasui Y Potter JD Colorectal adenomas and the C677T MTHFR polymorphism: evidence for gene-environment interaction? Cancer Epidemiol Biomarkers Prev 1999 8 659 668 10744125

[B47] Friso S Choi SW Girelli D Mason JB Dolnikowski GG Bagley PJ Olivieri O Jacques PF Rosenberg IH Corrocher R Selhub J A common mutation in the 5,10-methylenetetrahydrofolate reductase gene affects genomic DNA methylation through an interaction with folate status Proceedings of the National Academy of Sciences of the United States of America 2002 99 5606 5611 11929966 10.1073/pnas.062066299 PMC122817

[B48] Jacques PF Bostom AG Williams RR Ellison RC Eckfeldt JH Rosenberg IH Selhub J Rozen R Relation between folate status, a common mutation in methylenetetrahydrofolate reductase, and plasma homocysteine concentrations Circulation 1996 93 7 9 8616944 10.1161/01.cir.93.1.7

[B49] Ulvik A Ueland PM Fredriksen A Meyer K Vollset SE Hoff G Schneede J Functional inference of the methylenetetrahydrofolate reductase 677C > T and 1298A > C polymorphisms from a large-scale epidemiological study Human Genetics 2007 121 57 64 10.1007/s00439-006-0290-2 17115185

